# Biological Functions of the Secretome of *Neisseria meningitidis*

**DOI:** 10.3389/fcimb.2017.00256

**Published:** 2017-06-16

**Authors:** Jan Tommassen, Jesús Arenas

**Affiliations:** Department of Molecular Microbiology and Institute of Biomembranes, Utrecht UniversityUtrecht, Netherlands

**Keywords:** *Neisseria meningitidis*, secretome, autotransporters, two-partner secretion system, host-pathogen interactions, immune evasion, biofilms

## Abstract

*Neisseria meningitidis* is a Gram-negative bacterial pathogen that normally resides as a commensal in the human nasopharynx but occasionally causes disease with high mortality and morbidity. To interact with its environment, it transports many proteins across the outer membrane to the bacterial cell surface and into the extracellular medium for which it deploys the common and well-characterized autotransporter, two-partner and type I secretion mechanisms, as well as a recently discovered pathway for the surface exposure of lipoproteins. The surface-exposed and secreted proteins serve roles in host-pathogen interactions, including adhesion to host cells and extracellular matrix proteins, evasion of nutritional immunity imposed by iron-binding proteins of the host, prevention of complement activation, neutralization of antimicrobial peptides, degradation of immunoglobulins, and permeabilization of epithelial layers. Furthermore, they have roles in interbacterial interactions, including the formation and dispersal of biofilms and the suppression of the growth of bacteria competing for the same niche. Here, we will review the protein secretion systems of *N. meningitidis* and focus on the functions of the secreted proteins.

## Introduction

The gram-negative diplococcus *Neisseria meningitidis* is a commensal bacterium residing in the upper respiratory tract of humans. It is transmitted through aerosols and can asymptomatically be present in up to 30% of the population for long periods exceeding 2 years. It is a close relative of another commensal of the nasopharynx, *Neisseria lactamica*, and of the pathogen *Neisseria gonorrhoeae*, which causes infections of the urogenital system. Incidentally, also *N. meningitidis* is pathogenic when it crosses the epithelial barriers of the nasopharynx to reach the bloodstream causing septicemia. From there, it can cross the blood-brain barrier causing meningitis (Pace and Pollard, 2012; Takada et al., 2016). Meningococcal disease has an incidence rate that ranges from <1 to 1,000 cases per 100,000 per year with large geographical and temporal differences (Rouphael and Stephens, 2012). It is lethal in 10% of the cases and causes severe sequelae in 30–50% of the survivors, including neurologic disabilities, seizures, hearing or visual loss, or cognitive impairment (Pace and Pollard, 2012). The high morbidity and mortality of the disease has urged in the past decades the development of suitable vaccines.

Based on the structure of the capsular polysaccharide, *N. meningitidis* is divided into 13 serogroups, five of which (A, B, C, W, and Y) are responsible for the majority of meningococcal disease. Vaccines have been developed based on the capsular polysaccharides of serogroups A, C, W, and Y (Crum-Cianflone and Sullivan, 2016). However, the capsule of serogroup B is not immunogenic and, therefore, a capsule-based vaccine for this serogroup could not be developed. Vaccines based on outer-membrane vesicles (OMVs) have been designed against serogroup B strains, but their efficacy was restricted to determined geographic areas affected by certain clones (Bjune et al., 1991; Oster et al., 2005). In the quest for alternative vaccine candidates, new cell-surface-exposed and secreted proteins have been identified and extensively studied in recent years. These studies have resulted in two vaccine formulations, Bexsero® (4CMenB) and Trumenba® (MenB-FHbp), with an expected broader spectrum than OMV-based vaccines (Crum-Cianflone and Sullivan, 2016). In addition, they have provided extensive insights into the functions of the surface-exposed and secreted proteins and their role in the biology of *N. meningitidis*.

Although certain lineages of *N. meningitidis* are highly invasive and frequently associated with the disease, the bacteria have evolved to live in the host asymptomatically. After entering a host, the bacteria adhere to epithelial surfaces in the nasopharynx, where they form microcolonies (Sim et al., 2000). These structures resemble biofilms and help the bacteria to persist under adverse conditions. As a commensal, *N. meningitidis* has evolved different mechanisms to evade the host immune system and to compete with other members of the oral microbiome. It has to scavenge the environment for nutrients, which are restricted in this niche. In all these processes, various components of the secretome, i.e., the total of proteins that are secreted across the cell envelope to the cell surface or beyond, are implicated. It has been a decade since the last review of the secretome of *N. meningitidis* (van Ulsen and Tommassen, 2006). Since then, significant progress has been made in solving the functions of the secreted proteins. In this review, we first briefly describe the protein translocation systems that are relevant for the secretion of proteins in *N. meningitidis*. Then, we describe the current insights into the functions of the secreted proteins.

## Protein translocation systems in *N. meningitidis*

In Gram-negative bacteria, several conserved pathways have evolved for the translocation of proteins across the cell envelope into the extracellular milieu (Costa et al., 2015). Of these pathways, the autotransporter, two-partner secretion (TPS), and the type I secretion (T1S) systems are active in *N. meningitidis*. In addition, these bacteria use a recently discovered mechanism, which is dependent on a member of a protein family called Slam, for the cell-surface exposure of lipoproteins (Hooda et al., 2016). The T1S system (T1SS) mediates the secretion of substrates in one step from the bacterial cytoplasm across the two membranes into the milieu. In the other pathways, the substrates are first translocated across the inner membrane, after which the periplasmic intermediate is translocated across the outer membrane. To varying extent, these two-step pathways deploy general machinery for the localization of envelope proteins, i.e., the Sec and Tat systems for protein translocation across the inner membrane, the Lol system for shuttling lipoproteins across the periplasm, and the BAM system for the assembly of integral outer membrane proteins (OMPs). In this section, we briefly describe the protein transport systems that are relevant in *N. meningitidis* and emphasize what is known about these systems in this organism. It is noteworthy that fundamental research in *Neisseria* spp. has played a leading role in uncovering some of these systems, particularly the autotransporter mechanism, the BAM system, and the Slam-dependent pathway. Except for Sec and Tat, the other translocation systems have been studied at least to some extent in *N. meningitidis*. For more extensive descriptions of the pathways, we refer in each subsection to specialized recent reviews.

### Transport and assembly of integral OMPs

Integral OMPs are not part of the secretome as they are not translocated across the entire cell envelope. Therefore, their functions are not discussed in this review. However, since their translocation and assembly machinery is deployed by protein secretion systems, this machinery will be described here.

**Figure 1:**
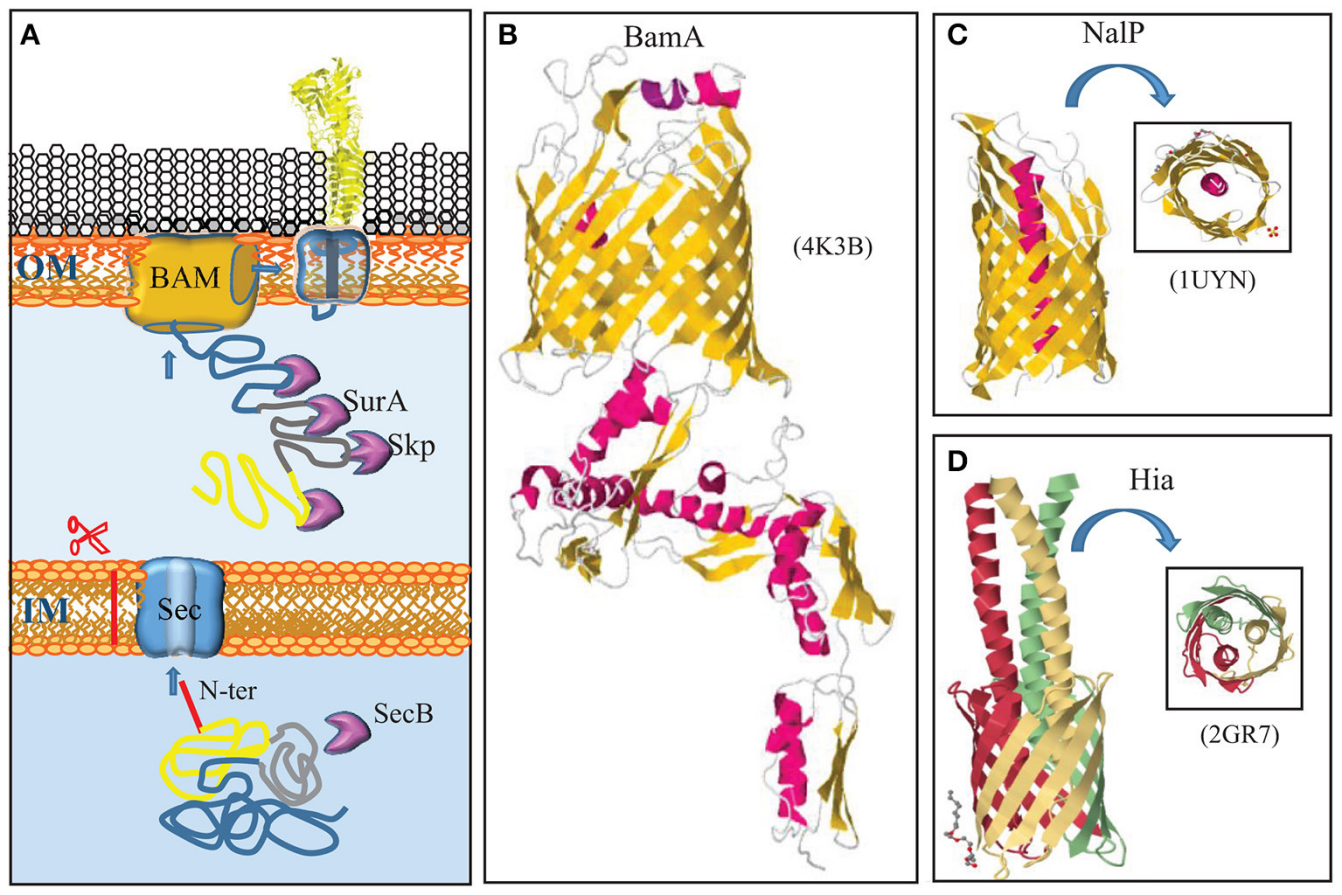
Autotransporter secretion system. **(A)** Autotransporters use the same machinery to reach the outer membrane (OM) as integral OMPs. They are synthesized with a cleavable N-terminal signal sequence (red) for translocation across the inner membrane (IM) via the Sec translocon. After periplasmic transit, escorted by chaperones like Skp and SurA, the C-terminal β-domain (blue) is integrated as a β-barrel into the OM by the BAM complex. Presumably during this integration, the passenger domain (yellow) is transported to the cell surface. A segment that connects the passenger with the β-domain (gray) forms an α-helix that plugs the channel within the barrel. **(B)** Structure of the central component of the BAM system, BamA, from *N. gonorrhoeae* (Noinaj et al., 2013). BamA is constituted of a β-barrel in the outer membrane and five periplasmic POTRA domains. α-Helices and β-strands are shown in red and yellow, respectively. **(C)** Structure of the β-domain of the autotransporter NalP with the α-helix that plugs the β-barrel and exposes the passenger, if connected, at the cell surface, indicated in red (Oomen et al., 2004). **(D)** Structure of the β-domains of the trimeric autotransporter Hia of *H. influenzae* (Meng et al., 2006). Each subunit is indicated with a different color. In panels **(C,D)**, a top view is shown in the insets. The structural data were retrieved from the Protein Data Bank (PDB) and access codes are provided in brackets. References are provided in the text.

The vast majority of integral OMPs consists of antiparallel amphipathic β-strands that form closed cylindrical structures, called β-barrels. They are synthesized in the cytoplasm as precursors with an N-terminal signal sequence, which marks them for transport across the inner membrane via the Sec system (von Heijne, 1990). The Sec system (for recent reviews, see Lycklama a Nijeholt and Driessen, 2012; Tsirigotaki et al., 2017) has, so far, not been studied in *N. meningitidis*, but homologs of all components have been identified in the available genome sequences (van Ulsen and Tommassen, 2006), and, therefore, it presumably functions similarly as in model organisms like *Escherichia coli*. In *E. coli*, the Sec system includes a chaperone, SecB, which prevents aggregation of client proteins in the cytoplasm and guides them to the inner membrane components of the system (Figure 1A). In the inner membrane, the heterotrimeric SecYEG complex forms the protein-translocating channel. Energy for transport is provided by the motor protein SecA, which hydrolyzes ATP, and by the proton-motive force, which is coupled to the translocation process presumably by the SecDF-YajC complex (Tsukazaki et al., 2011). After passage of the precursor through the Sec translocon, its signal sequence is removed by the signal peptidase LepB (Auclair et al., 2012).

All integral OMPs studied so far use the Sec system for translocation across the inner membrane. The channel in the SecYEG translocon is narrow and only allows for the translocation of completely unfolded proteins in a linear fashion. Some proteins have to be exported in a folded form, e.g., because they bind a co-factor in the cytoplasm. Such proteins share a consensus “twin-arginine” motif ([S/T]-R-R-x-F-L-K) in the N-terminal region of their signal sequences, and they use an alternative apparatus, the twin-arginine translocation (Tat) system, to cross the inner membrane (for recent reviews, see Palmer and Berks, 2012; Patel et al., 2014). The Tat system of *E. coli* consists of three proteins, TatA, TatB, and TatC, all of which are also present in *N. meningitidis* (van Ulsen and Tommassen, 2006).

Once OMPs are released from the Sec machinery, chaperones, such as SurA and Skp, bind the mature proteins to prevent their aggregation in the periplasm (Figure 1A). In *E. coli*, SurA appears to be the main periplasmic chaperone for OMPs as its depletion drastically reduces the OMP content, whereas depletion of Skp had only minor effects (Sklar et al., 2007). In sharp contrast, mutational analysis indicated that SurA has no appreciable role in OMP biogenesis in *N. meningitidis*, whereas deletion of *skp* affected the major OMPs, i.e., the porins (Volokhina et al., 2011). Also the protease DegP has been suggested to have a role as a periplasmic chaperone in OMP biogenesis (Sklar et al., 2007; Krojer et al., 2008). However, its primary role in this process presumably is the degradation of toxic and membrane-damaging misfolded OMPs in the periplasm (Ge et al., 2014). Inactivation of DegQ, the DegP homolog of *N. meningitidis*, did not have any appreciable effect on OMP biogenesis (Volokhina et al., 2011).

After transit of the periplasm, OMPs are assembled into the outer membrane by the β-barrel assembly machinery (BAM) (Figure 1A) (for a recent review, see Noinaj et al., 2017). The central component of the BAM was originally discovered by Voulhoux et al. (2003) in *N. meningitidis*. This protein, previously known as Omp85 and now called BamA, is essential for bacterial viability and homologs are found in all Gram-negative bacteria and even in mitochondria, where it performs a similar function (Walther et al., 2009). The first complete BamA crystal structure solved was that of *N. gonorrhoeae* (Noinaj et al., 2013). The protein consists of a C-terminal 16-stranded β-barrel, which is inserted into the outer membrane, and five repeated polypeptide transport-associated (POTRA) domains extending into the periplasm (Figure 1B). In *E. coli*, BamA forms a complex with four lipoproteins, BamB, C, D, and E. These lipoproteins are less conserved, and only BamD is essential (Ricci and Silhavy, 2012; Noinaj et al., 2017). The BAM of *N. meningitidis* lacks the BamB component, but it contains the peptidoglycan-associated OMP RmpM, which stabilizes the complex (Volokhina et al., 2009). Also in *N. meningitidis*, BamD is essential (Volokhina et al., 2009), but a *bamD* mutant (originally described as *comL* mutant) of *N. gonorrhoeae* appeared to be viable (Fussenegger et al., 1996).

### Autotransporter secretion

The first autotransporter ever described is the IgA protease of *N. gonorrhoeae* (Pohlner et al., 1987). It is a classical autotransporter that consists of an N-terminal signal sequence for transport across the inner membrane via the Sec system, a secreted passenger domain, and a C-terminal β-domain. The β-domain was suggested to insert as a β-barrel into the outer membrane to form a pore through which the passenger domain is secreted without assistance of any other proteins, hence the name autotransporter. The first crystal structure of such a β-domain that was solved, i.e., that of the autotransporter NalP of *N. meningitidis*, seemed to confirm this idea (Oomen et al., 2004). This structure revealed a 12-stranded β-barrel with an internal hydrophilic pore filled by an N-terminal α-helix that would expose the passenger, if connected, at the cell surface (Figure 1C). However, it was also noticed that the internal pore was very narrow, probably too narrow to allow for the passage of a linear polypeptide that contains even small structural elements such as oligopeptide loops formed by disulfide bonds.

It is clear now that the name autotransporter is an oversimplification of the secretion pathway that is used. Autotransporters largely utilize for their secretion the machinery that is used by OMPs (Figure 1A) (for a recent review, see Grijpstra et al., 2013). First, they are translocated across the inner membrane by the Sec machinery. In the periplasm, they are escorted by chaperones, such as SurA, Skp, and DegP (Purdy et al., 2007; Ieva and Bernstein, 2009; Ruiz-Perez et al., 2009), although inactivation of the corresponding genes had no noticeable effect on autotransporter biogenesis in *N. meningitidis* (Volokhina et al., 2011). Also the BAM complex in the outer membrane is required for autotransporter biogenesis (Voulhoux et al., 2003), presumably for the insertion of the β-domain but possibly also for the translocation of the passenger domain, as a passenger stalled in the translocation process could be cross-linked to BamA (Ieva and Bernstein, 2009). It was proposed that the β-domain of the autotransporter and the β-barrel of BamA form a hybrid barrel with an internal diameter that is wide enough to allow for the translocation of the passenger, even if it contains small folded domains (Ieva et al., 2011). Protein folding at the cell surface probably provides the energy source to drive translocation (Drobnak et al., 2015). After outer membrane translocation, the passenger domain can remain attached to the β-domain, or it can be released into the extracellular milieu by one of several proteolytic mechanisms (Grijpstra et al., 2013). For the mechanisms relevant in *N. meningitidis*, we refer to the description of the individual autotransporters below. Besides the BAM complex, also the TAM complex may play a role in the secretion of a subset of autotransporters (Selkrig et al., 2012). The TAM complex consists of an OMP, TamA, which is a homolog of BamA, and an inner membrane protein TamB, which spans the periplasm and interacts with TamA. The precise role of the TAM complex is unclear. Reconstitution experiments showed that the BAM complex and chaperone SurA were necessary and sufficient to mediate the translocation of an autotransporter into proteoliposomes (Roman-Hernandez et al., 2014). Perhaps, TAM can substitute for BAM for some autotransporters. The role of TAM in autotransporter secretion has not been investigated in *N. meningitidis*.

Among autotransporters, four subcategories can be distinguished (Grijpstra et al., 2013). Two of them are present in *N. meningitidis*, the classical autotransporters, which include IgA protease and NalP, and the trimeric autotransporters. In trimeric autotransporters, the β-domains of each subunit contribute four β-strands to form a similar 12-stranded β-barrel as does the β-domain of the classical autotransporters (Figure 1D) (Meng et al., 2006). The structure of the passenger domain, however, is entirely different, as will be discussed further below.

### Two-partner secretion (TPS) system

**Figure 2:**
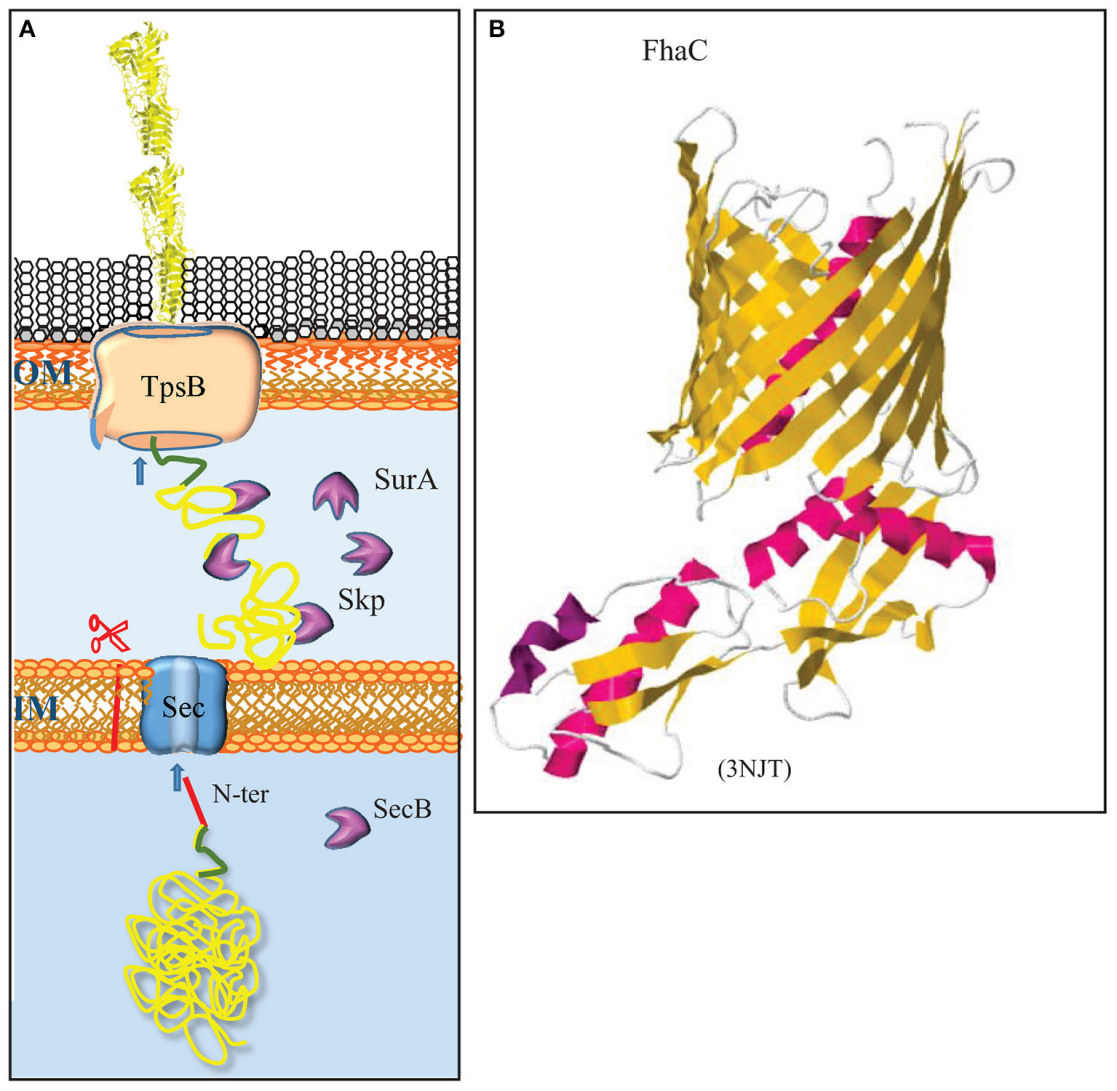
The two-partner secretion system. **(A)** The TpsA substrates contain a cleavable signal sequence (red) for Sec-mediated translocation across the inner membrane (IM). After chaperone-escorted transit of the periplasm, the N-terminal TPS domain (green) interacts with the integral OMP TpsB, which mediates transport across the outer membrane (OM). **(B)** Structure of the TpsB FhaC of *B. pertussis* (Clantin et al., 2007). TpsB consists of a β-barrel domain embedded in the OM and two periplasmic POTRA domains that interact with the TPS domain of substrates. The first POTRA domain is connected via a linker with an N-terminal α-helix that plugs the β-barrel in the resting state. The structure of the R450A mutant of FhaC is depicted to show the plugging of the channel. β-Strands and α-helices are colored yellow and red, respectively. The PDB access code is provided in brackets.

The TPS system facilitates the secretion of very large β-helical proteins with often virulence-related functions (for a recent review, see Jacob-Dubuisson et al., 2013). The two partners are the secreted protein, which is generically called TpsA, and a dedicated transporter in the outer membrane called TpsB (Figure 2A). TpsB is a homolog of BamA. From the C to the N terminus, it consists of a 16-stranded β-barrel, two POTRA domains at the periplasmic side, and an α-helix that plugs the channel in the barrel (Figure 2B) (Clantin et al., 2007). TpsA is transported across the inner membrane by the Sec system and escorted in the periplasm by chaperones (Jacob-Dubuisson et al., 2013). The mature TpsA contains a conserved TPS domain near the N terminus that is recognized by the POTRA domains of TpsB (Figure 2B). This interaction leads to removal of the plug helix and further conformational changes in TpsB (Maier et al., 2015), allowing for the transport of TpsA to the cell surface via the barrel of TpsB. *N. meningitidis* strains generally contain at least one conserved locus with *tps* genes, but genome sequence analysis indicated that various strains can contain up to five different *tpsA* genes and two *tpsB* genes (van Ulsen and Tommassen, 2006; van Ulsen et al., 2008). One of the TpsB proteins was shown to have broad substrate specificity, whereas the other one was specific for the TpsA encoded by the same operon (ur Rahman and van Ulsen, 2013). Substrate specificity was shown to be determined by the POTRA domains (ur Rahman et al., 2014).

### Transport of lipoproteins to the outer membrane

**Figure 3:**
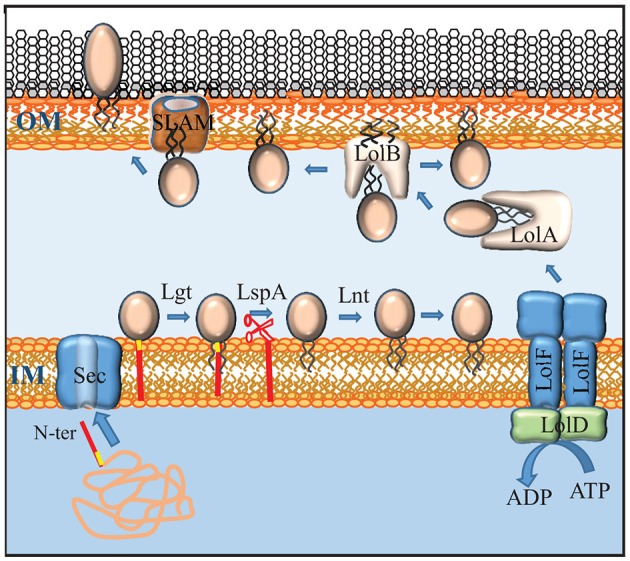
Maturation, transport and secretion of lipoproteins. Lipoproteins contain a lipobox (yellow) at the C terminus of their signal sequence (red). After transport across the inner membrane (IM) by the Sec system, the lipoprotein precursor is modified by the addition of a diacylglyceryl moiety to the sulfhydryl group of the cysteine in the lipobox by Lgt, followed by cleavage of the signal sequence by LspA, and the addition of a third, amide-linked fatty acyl chain by Lnt. Lipoproteins that don't contain an IM-retention signal (also called Lol-avoidence motif) are expelled from the IM by an ABC transporter that, in *Neisseria*, consists of a dimer of LolF (instead of the LolCE heterodimer found in *E. coli*) and a dimer of LolD. The chaperone LolA shields the lipid moiety of the lipoprotein during periplasmic transit and shuttles it to the receptor LolB that inserts it into the inner leaflet of the OM. Some lipoproteins are translocated to the outer face of the OM via a mechanism that requires a SLAM protein. Whether SLAM forms the actual translocation channel itself is not known.

Besides integral OMPs, the outer membrane contains lipoproteins, which are peripherally attached to the membrane via an N-terminal lipid moiety. Lipoproteins are synthesized as precursors with an N-terminal signal sequence for export via the Sec or Tat machinery. Their signal sequences contain at the C terminus a conserved motif, [LVI][ASTVI][GAS]C, called lipobox, where the conserved cysteine represents the first residue of the mature protein (Hayashi and Wu, 1990). After translocation across the inner membrane, this cysteine is modified (for a recent review, see Buddelmeijer, 2015). First, a diacylglycerol moiety derived from phosphatidylglycerol is covalently attached to the sulfhydryl group of the cysteine by the enzyme lipoprotein diacylglyceryl transferase (Lgt) (Figure 3). Then, the signal sequence is cleaved by the dedicated lipoprotein signal peptidase LspA, after which the α-amino group of the cysteine is acylated by the enzyme apolipoprotein N-acyltransferase (Lnt) (Figure 3). In *E. coli*, these three enzymes are essential. However, the *lnt* gene could be inactivated in *N. meningitidis* and *N. gonorrhoeae* (LoVullo et al., 2015; da Silva et al., 2016). As the outer membrane contains essential lipoproteins, such as BamD (see above), this implies that these *Neisseria* spp., in contrast to *E. coli*, can transport diacylated lipoproteins to the outer membrane, which was indeed demonstrated in *N. meningitidis* (da Silva et al., 2016).

After their maturation, lipoproteins can remain attached to the inner membrane or be transported to the outer membrane. In *E. coli*, the destination of the lipoprotein is determined by the identity of the amino-acid residue directly adjacent to the lipidated N-terminal cysteine (Yamaguchi et al., 1988). Basically, an aspartate residue at the +2 position functions as an inner-membrane-retention signal, whilst lipoproteins destined to outer membrane have a different residue at this position. However, this signal appears to be species specific as other sorting rules were reported for lipoproteins in other bacteria (Schulze and Zückert, 2006; Narita and Tokuda, 2007). The sorting rules for lipoproteins in *N. meningitidis* have not been determined, but the simple +2 rule of *E. coli* does not seem to apply as a DsbA lipoprotein with a serine at the +2 position was experimentally demonstrated to localize to the inner membrane (Tinsley et al., 2004).

Lipoproteins are targeted to the periplasmic side of the OM by the Lol system. This system consists of an ATP-binding cassette (ABC) transporter LolCDE in the inner membrane, a periplasmic chaperone LolA, and LolB, which functions as a receptor in the outer membrane (Figure 3) (for a recent review, see Narita and Tokuda, 2016). The LolCDE complex consists of a heterodimer of two homologous integral membrane proteins, LolC and LolE, which is associated with a dimer of the cytoplasmic ABC protein LolD. This complex selects the substrate lipoproteins that don't carry an inner-membrane retention signal and provides the energy to release them from the inner membrane by ATP hydrolysis (Yakushi et al., 2000). Interestingly, *N. meningitidis* and *N. gonorrhoeae*, as well as many other Gram-negative bacteria, each contain only a single protein that is homologous to LolC and LolE and that has specific characteristics of both of them (Figure 3) (LoVullo et al., 2015). This protein, which is called LolF, presumably forms a homodimer in the inner membrane, and it was suggested that the presence of a LolF instead of LolCE is related to the capacity of these bacteria to transport diacylated lipoproteins, i.e., lipoproteins not modified by Lnt, to the outer membrane (LoVullo et al., 2015). After release from the inner membrane, the lipoprotein is captured by the chaperone LolA, which shields its hydrophobic lipid moiety from the aqueous environment of the periplasm, thus forming a water-soluble complex (Matsuyama et al., 1995). From LolA, the lipoprotein is transferred to LolB, which is a structural homolog of LolA. LolB is a lipoprotein itself and it is attached with its lipid moiety in the OM (Figure 3). LolB then mediates the insertion of the lipoprotein into the inner leaflet of the OM (Matsuyama et al., 1997).

### Transport of lipoproteins to the cell surface

How lipoproteins are translocated across the outer membrane to reach the cell surface has only been studied in few exceptional cases but is an emerging field of research (for a recent review, see Wilson and Bernstein, 2017). In the best studied cases, the lipoproteins utilize conserved protein secretion systems to reach the cell surface, such as the type 2 secretion system (T2SS) (for a review, see Nivaskumar and Francetic, 2014), a system that is related to the machinery required for the biogenesis of type IV pili but is not operational as a protein secretion system in *N. meningitidis*. In this case, the lipoproteins contain an inner membrane retention signal to avoid the Lol system after their transport across the inner membrane via Sec or Tat (Pugsley and Kornacker, 1991; Putker et al., 2013). These lipoproteins are directly secreted from the periplasmic side of the inner membrane to the cell surface using the T2S machinery. Other lipoproteins, such as NalP of *N. meningitidis* (van Ulsen et al., 2003), are autotransporters. Before translocation across the outer membrane, these autotransporter lipoproteins presumably use the Lol system to reach the inner face of the outer membrane, although this has experimentally not yet been proven. Recently, a new general pathway dedicated to the transport of lipoproteins to the cell surface was uncovered in *N. meningitidis* (Hooda et al., 2016). This pathway is an extension of the Lol pathway as the substrates appear to use the Lol system to reach the outer membrane. A family of proteins named surface lipoprotein assembly modulator (Slam) was found to be involved in the subsequent translocation across the outer membrane (Figure 3). Slam proteins consist of an N-terminal periplasmic domain containing two tetratricopeptide repeats and a C-terminal 14-stranded β-barrel embedded in the outer membrane. There is no homolog of Slam proteins in *E. coli*, and the synthesis of cell-surface-exposed lipoproteins from *N. meningitidis* in *E. coli* does not lead to their cell-surface exposition. However, when Slam was co-expressed, these lipoproteins were transported to the *E. coli* cell surface (Hooda et al., 2016). Whether Slam forms the actual translocation channel for the lipoproteins or guides them to an alternative translocation apparatus, such as BAM, remains to be investigated. *N. meningitidis* has two Slam proteins with different substrate specificity. Slam1 has broad specificity and is involved in the translocation of at least the transferrin-binding protein B (TbpB), the lactoferrin-binding protein B (LbpB), and the factor H-binding protein (fHbp). Slam2 appears to be specific for hemoglobin-haptoglobin utilization protein A (HpuA). Interestingly, these lipoproteins, as well as another cell-surface-exposed lipoprotein, i.e., neisserial heparin-binding antigen (NHBA), show considerable structural similarity (see below), which may reflect a prerequisite for Slam-dependent transport and/or a common evolutionary origin. Slam homologs are widely distributed among proteobacteria (Hooda et al., 2016), suggesting that they represent a new general pathway dedicated to the secretion of lipoproteins. It is noteworthy, however, that TbpB expressed in *N. gonorrhoeae* without its lipid moiety was secreted into the culture supernatant (Ostberg et al., 2013), suggesting that the Slam-dependent pathway may have broader specificity than just lipoproteins.

### Type 1 secretion system (T1SS)

**Figure 4:**
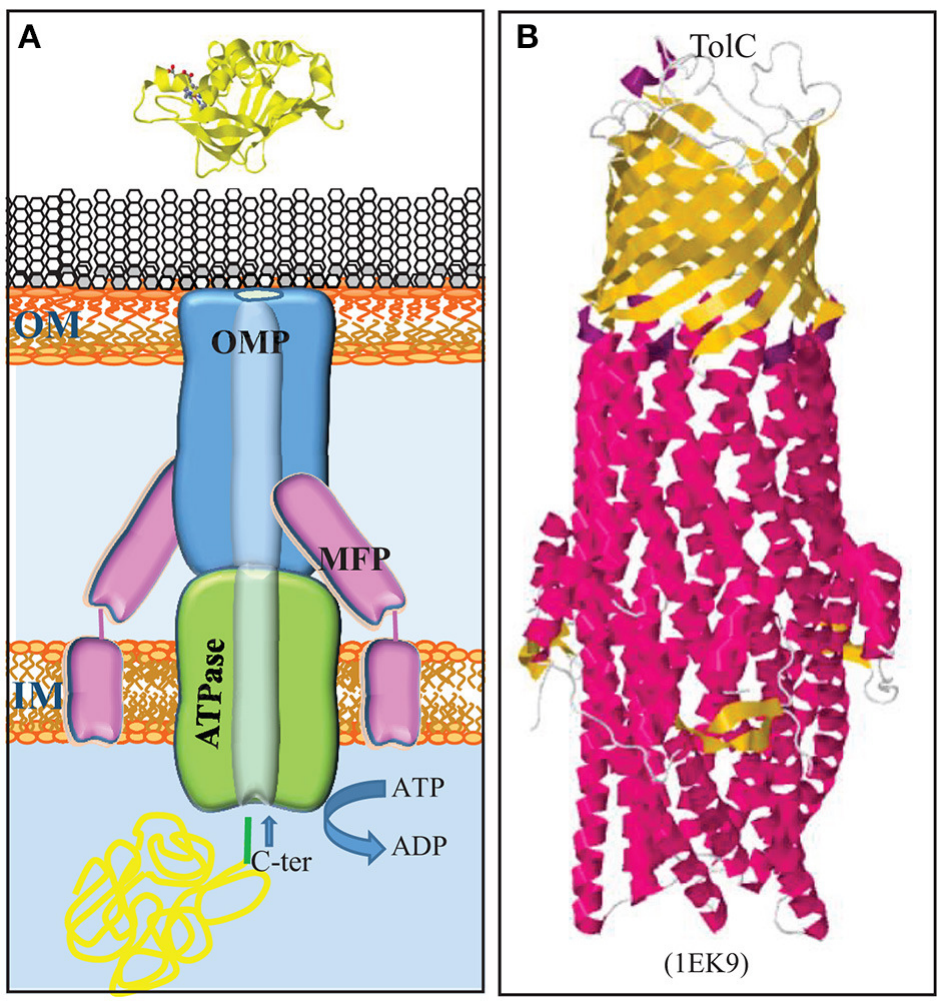
Type 1 secretion system. **(A)** The T1SS consists of three proteins, a trimeric OMP (blue), an ABC transporter in the inner membrane (IM) (green), and an IM-anchored membrane-fusion protein (MFP) (pink), which together form a translocation channel for substrates directly from the bacterial cytoplasm to the external milieu. Substrates contain a secretion signal at their C terminus (green) for recognition by the machinery. **(B)** Structure of the homotrimeric OM T1SS component of *E. coli*, TolC (Koronakis et al., 2000). The trimer consists of a β-barrel in the OM and long α-helices, which form a tunnel that deeply penetrates into the periplasm. β-Strands and α-helices are colored yellow and red, respectively. The PDB access code is given in brackets.

The T1SS is composed of three proteins: an ABC protein in the inner membrane, a TolC-type channel protein in the outer membrane, and a membrane-fusion protein that connects the integral inner and outer membrane components (Figure 4A) (for a recent review, see Lenders et al., 2013). The outer membrane component is trimeric protein that forms a 12-stranded β-barrel in the outer membrane to which each protomer contributes four β-strands (Figure 4B). At the periplasmic side, the β-strands are connected by long α-helices that form together a long hollow conduit that spans the periplasm and reaches the ABC transporter in the inner membrane (Koronakis et al., 2000). This machinery mediates the secretion of substrates directly from the cytoplasm into the extracellular milieu without a periplasmic intermediate (Figure 4A). Thus, these substrates do not contain an N-terminal signal sequence for recognition by the Sec or Tat systems. Instead, they contain a recognition signal at the C terminus to interact with the ABC component of the T1SS. Many T1SS substrates belong to the RTX protein family, where RTX stands for repeats in toxin (Linhartová et al., 2010). These proteins contain glycine-rich repeats with the consensus sequence GGxGxDxxx (where x is any amino acid) near the secretion signal. These repeats bind calcium ions, which promotes folding in the extracellular medium where, in contrast to the cytoplasm, the concentration of calcium is high. Thus, besides ATP hydrolysis and the proton-motive force, extracellular folding might provide a driving force for translocation (Lenders et al., 2013). The components constituting the T1SS of *N. meningitidis* have been identified (Wooldridge et al., 2005).

## Structure and function of secreted proteins

Table 1 summarizes the functions of the secreted proteins that are discussed in detail below.

**Table 1:** Secretion mechanism and functions of secreted proteins.

**Protein**	**Secretion system^a^^,^^b^**	**Biological functions^c^**
TbpB	Slam	Iron acquisition from transferrin
LbpB	Slam	Iron acquisition from lactoferrin
		Protection against lactoferricin
HpuA	Slam	Heme iron acquisition from hemoglobin(-haptoglobin)
fHbp	Slam	Prevention of complement activation by binding factor H
		Protection against host defense peptide LL-37
NHBA	Slam?	Serum resistance by binding heparin
		Adhesion by binding heparan sulfate proteoglycans
		Initiation of biofilm formation
		Increase of endothelial permeability
IgA protease	Classical AT	Cleavage of IgA and other host proteins
		Immune modulation by cleavage transcription factor NF-κB
		Initiation of biofilm formation
		Binding heparin
NalP	Classical AT	Proteolytic release of bacterial cell-surface-exposed proteins Cleavage of complement factor C3
App, AusI	Classical AT	Binding of histones and cleavage of histone H3
		Induction of apoptosis
		Adhesion
AutA	Classical AT	Autoaggregation
		Stimulation of CD4^+^ T-cells and B-cells
AutB	Classical AT	Biofilm formation
NadA	Trimeric AT	Adhesion and invasion
		Binding Hsp90
NhhA	Trimeric AT	Adhesion
		Binding laminin and heparan sulfate
		Serum resistance by binding vitronectin
		Apoptosis of macrophages
		Immune modulation by affecting differentiation monocytes
TpsA	TPS	Interbacterial competition
		Intracellular survival
		Adhesion
		Biofilm formation
FrpC	T1SS	Adhesion
FrpD	Slam?	Adhesion
MafB	Unknown	Interbacterial competition
TspB	Unknown	Binding immunoglobulins
		Biofilm formation

a *Question marks indicate that suggested secretion mechanism is not demonstrated*.

b *AT, autotransporter*.

c *For references, see text*.

### Cell-surface-exposed lipoproteins

In this section, we discuss the structure and function of five well-characterized cell-surface-exposed lipoproteins. Two other such lipoproteins, i.e., the autotransporter NalP and FrpD, which has a role in the T1SS, are discussed in subsequent sections. Several other lipoproteins have been suggested in the literature to be exposed at the cell surface in *N. meningitidis*. However, even if their function is known, it is not clear why they are transported to the cell surface to exert such function, and in some cases, their function is even incompatible with surface localization. We don't discuss these lipoproteins here and feel that their localization should be further investigated. For a discussion about caveats and pitfalls in assessing lipoprotein localization, see Wilson and Bernstein (2017).

#### Lipoproteins involved in iron acquisition: TbpB, LbpB, and HpuA

One of the primary defense mechanisms of a host against invading bacterial pathogens is to deprive them from essential nutrients, such as iron, a defense mechanism that is known as nutritional immunity. In the human host, iron is bound by proteins, such as transferrin in serum, lactoferrin in secretions and mucosal surfaces, and hemoglobin and ferritin within cells. These proteins have very high affinities for iron, and, consequently, the concentration of free iron in the human fluids is too low to support microbial growth (Weinberg, 2009). Many bacteria produce siderophores, which are low-molecular-weight iron chelators that sequester otherwise inaccessible ferric iron from the environment. However, *Neisseria* spp. do not secrete siderophores; instead, they synthesize receptors that directly hijack iron sequestered in the iron-binding proteins of the host (Schryvers and Stojiljkovic, 1999). Several of these receptors consist of two proteins, a surface-exposed lipoprotein and an integral OMP. The integral OMP belongs to the TonB-dependent family (Tdf) of receptors, which includes also siderophore receptors. These receptors form 22-stranded β-barrels that are closed by an N-terminal plug. They interact with the TonB complex in the inner membrane, which deploys the proton-motive force to energize transport across the outer membrane (for a review, see Noinaj et al., 2010).

**Figure 5:**
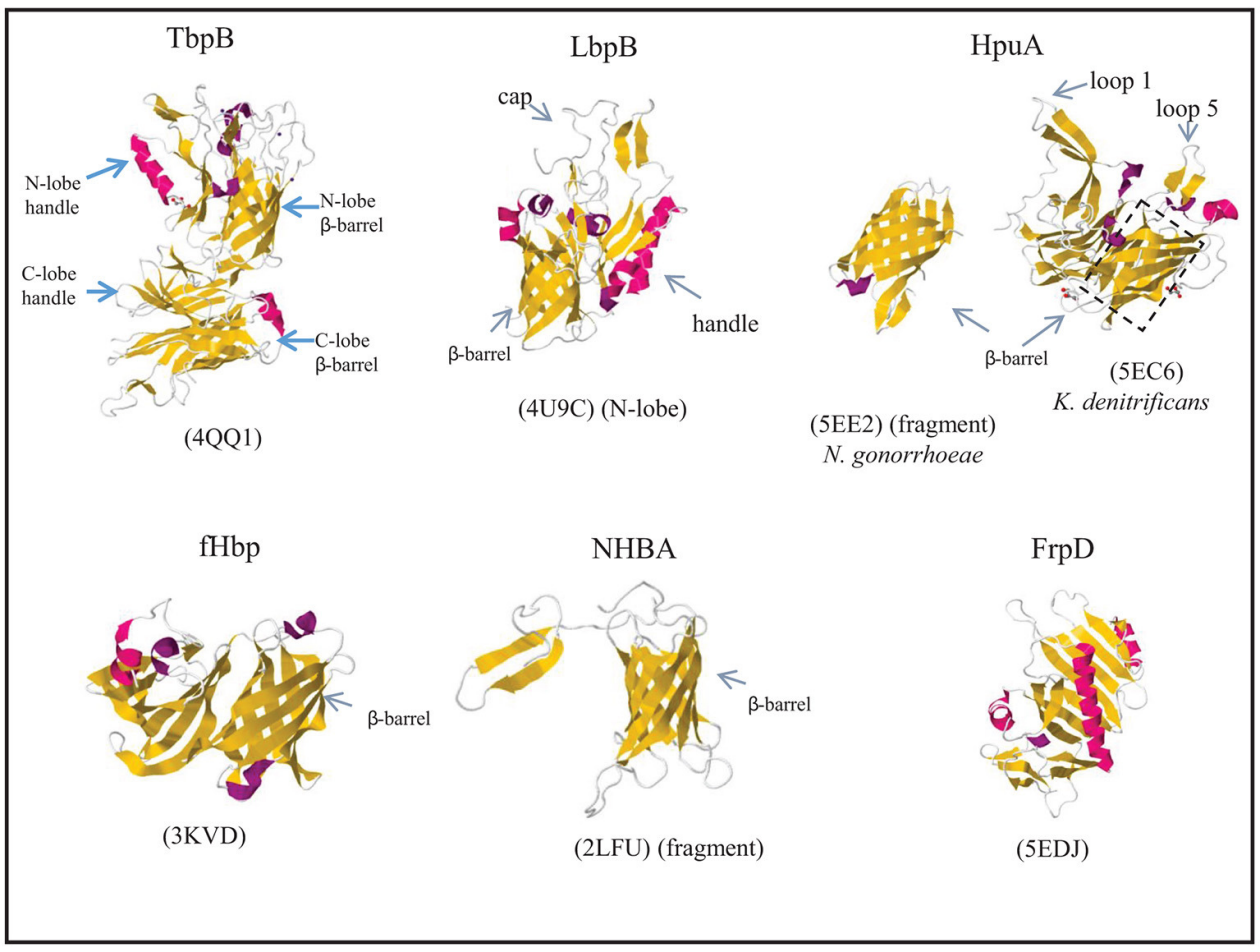
Structures of surface-exposed lipoproteins in *N. meningitidis*. Where indicated, the structure of only a fragment of the complete protein is available. The structure of *N. meningitidis* HpuA has not been solved; instead, those of *K. denitrificans* HpuA and a C-terminal fragment of *N. gonorrhoeae* HpuA are shown. β-Strands and α-helices are colored yellow and red, respectively, and relevant domains of each protein are indicated. The PDB access codes are given in brackets and references are provided in the text.

The transferrin receptor in *Neisseria* spp. consists of the Tdf member TbpA and the surface-exposed lipoprotein TbpB. The synthesis of these proteins is induced under iron limitation. In *N. meningitidis*, both proteins are essential for the acquisition of iron from human transferrin (Irwin et al., 1993), whilst TbpB is dispensable in *N. gonorrhoeae*, although its presence considerably enhances the efficiency of the process (Anderson et al., 1994). Both proteins can bind transferrin, but, in contrast to TbpA, TbpB preferably binds the iron-loaded form (Cornelissen and Sparling, 1996; Boulton et al., 1998). Thus, TbpB may enhance iron acquisition by selecting holo-transferrin to bind to the receptor. In addition, TbpB's affinity for the holo form may help in the release of the apo form from the receptor once iron is delivered. TbpB is a bilobed protein probably resulting from gene duplication. Each lobe consists of a β-barrel domain and an adjacent handle domain, which is also rich in β-sheet structure (Figure 5) (Calmettes et al., 2012; Noinaj et al., 2012). Human transferrin is also a bilobed protein with a Fe^3+^-binding site within each lobe (Hall et al., 2002). Structural analysis revealed that TbpA and TbpB both bind the C-lobe of transferrin using non-overlapping binding sites (Noinaj et al., 2012). The C-lobe of transferrin consists of two subdomains, C1 and C2, which are connected by a hinge at the base of a deep cleft that contains the Fe^3+^-binding site. TbpB binds both the C1 and C2 subdomains of holo-transferrin via the handle and β-barrel domains in its N-lobe, respectively (Calmettes et al., 2012). This explains the selectivity of TbpB for holo-transferrin as the apo-form shows a 51° rotation of the C2 domain which would drastically reduce the TbpB-transferrin interface (Calmettes et al., 2012). In the available structures, the C-lobe of TbpB is not involved in transferrin binding, and its function is unclear. An α-helix from extracellular loop 3 of TbpA intrudes into the cleft between the C1 and C2 subdomains of transferrin, which induces a partial opening of the cleft and destabilizes the coordination site of the ferric ion (Noinaj et al., 2012), which facilitates its release. Released iron would enter a closed chamber formed by TbpA, TbpB, and transferrin, which prevents its diffusion into the medium and positions it for further transport through the barrel of TbpA (Noinaj et al., 2012).

The lactoferrin receptor is constituted by the Tdf member LbpA and the surface-exposed lipoprotein LbpB (Pettersson et al., 1994b, 1998; Prinz et al., 1999). Whilst the corresponding genes are ubiquitous in *N. meningitidis*, they are disrupted in about half of the *N. gonorrhoeae* isolates (Anderson et al., 2003). Nevertheless, expression of a functional lactoferrin receptor in *N. gonorrhoeae* provided a competitive advantage in a human infection model, and it was essential for infection by a strain lacking the transferrin receptor (Anderson et al., 2003). LbpA and LbpB both bind lactoferrin, but LbpB is not essential for its utilization as an iron source in both species (Pettersson et al., 1998; Biswas et al., 1999). Whilst lactoferrin and transferrin are homologous proteins, also LbpA and LbpB share extensive sequence similarity with TbpA and TbpB, respectively (Pettersson et al., 1994a, 1998). Crystallization of the N lobe of LbpB revealed a structure very similar to that of the corresponding part of TbpB. It also consists of two domains, a handle domain and a β-barrel domain with long loops projecting from both domains and forming an extended cap region (Figure 5) (Brooks et al., 2014). *In silico* docking experiments indicated that the cap region constitutes the binding site to which lactoferrin binds with its N-lobe (Brooks et al., 2014). However, recent biochemical analysis revealed that it preferentially binds the C-lobe of iron-loaded lactoferrin (Ostan et al., 2017), just like TbpB binds the C-lobe of iron-loaded transferrin (see above), suggesting a similar role for LbpB as for TbpB in selecting iron-loaded ligands. LbpB appears to have an additional role in protecting the bacteria against the bactericidal activity of lactoferricin (Morgenthau et al., 2012). Lactoferricin is a small cationic peptide, which is released from the N terminus of lactoferrin by proteolysis (Bellamy et al., 1992; Gifford et al., 2005). LbpB contains two highly variable stretches rich in negatively charged amino-acid residues in its C-lobe (Pettersson et al., 1999) that have been shown to mediate protection against this cationic peptide (Morgenthau et al., 2014). Thus, binding of the N-lobe of lactoferrin to the C-lobe of LbpB may prevent proteolysis events that generate lactoferricin. In addition or alternatively, the C-lobe may also be able to bind the free peptide, thus neutralizing its toxic effects (Ostan et al., 2017).

*N. meningitidis* can express two receptors for hemoglobin, HmbR and HpuAB, which enable it to extract heme from hemoglobin and use it as an iron source (Stojiljkovic et al., 1995; Lewis et al., 1997). Of these, the HpuAB system is also capable of extracting heme from hemoglobin-haptoglobin complexes (Lewis et al., 1997). Expression of the corresponding genes is controlled by iron availability and is prone to phase variation by slipped-strand mispairing (Lewis et al., 1999). HpuAB is a bipartite receptor, in which HpuB is a Tdf member and HpuA is a surface-exposed lipoprotein. In the absence of HpuA, HpuB still binds its ligands but with low affinity, and they cannot be used as an iron source (Rohde et al., 2002; Rohde and Dyer, 2004). Ligands did not bind to cells expressing only HpuA (Rohde and Dyer, 2004), but a direct interaction could be demonstrated in pull-down assays with purified HpuA (Wong et al., 2015). The crystal structure was solved of *Kingella denitrificans* HpuA alone and in complex with hemoglobin, and of a C-terminal fragment of *N. gonorrhoeae* HpuA (Wong et al., 2015). HpuA is about half the size of TbpB, and its structure shows a similar fold as a single lobe of TbpB (Figure 5). It consists of a compact C-terminal β-barrel and an N-terminal open β-sandwich domain. Two large loops, loops 1 and 5, extend from the core of the protein. These exposed loops show high sequence variability, suggesting they are under immune selection. In spite of their solvent exposure, these loops have a high content of hydrophobic amino-acid residues, and although they display high sequence variability, they are the major interaction sites of hemoglobin, with loops 1 and 5 contacting the β- and α-chains, respectively, of a hemoglobin dimer. Additional interactions involve residues on two other loops.

#### Factor H binding protein (fHbp)

The complement system plays a key role in the host's defense against microbial invaders. Host cells are protected from complement activation, amongst others by binding the soluble factor H, a major negative regulator of the alternative complement pathway. By producing fHbp (a.k.a. GNA1870 or LP2086), *N. meningitidis* hijacks factor H and thereby limits complement activation at its surface (Madico et al., 2006; Schneider et al., 2006). Indeed, fHbp expression was found to improve bacterial survival in human blood and serum (Seib et al., 2009). Additionally, fHbp synthesis was found to protect the bacteria against the cationic antimicrobial peptide LL-37, a host defense peptide that destabilizes negatively charged bacterial membranes (Seib et al., 2009). Presumably, LL-37 interacts with fHbp also by electrostatic interaction.

fHbp is highly immunogenic and induces bactericidal antibodies that activate the classical complement pathway. In addition, antibodies may prevent binding of factor H to fHbp and thereby prevent inhibition of the alternative pathway. Based on sequence variation, two different subfamilies (Fletcher et al., 2004) or three variant groups (Masignani et al., 2003) were distinguished, with very limited immunological cross-reactivity between the groups (Masignani et al., 2003). One and two variants are included as components in the recently developed vaccines Bexsero® and Trumenba®, respectively.

The level of expression of fHbp varies considerably between different strains (Biagini et al., 2016), which may affect vaccine efficacy. Synthesis of the protein is induced under oxygen limitation (Oriente et al., 2010). Furthermore, fHbp synthesis is post-transcriptionally controlled by temperature due to the presence of a secondary structure at the 5′ end of its mRNA, which could function as a thermosensor; expression is considerably lower at 30°C than at 37°C (Loh et al., 2016). Thus, the synthesis of fHbp is presumably low on the mucosal surfaces of the nasopharynx, where the temperature is relatively low and the oxygen concentration is high. Consequently, vaccines solely based on fHbp will possibly not prevent carriage and spreading of the bacteria amongst the population and offer limited herd immunity (Loh et al., 2016). The synthesis of fHbp will increase as the bacteria reach the submucosal epithelial surfaces and the bloodstream, i.e., when protection against the host's defense mechanisms becomes essential. The synthesis of fHbp is also influenced by iron availability. However, whilst expression is reduced under iron limitation in most strains, it is increased in some lineages (Sanders et al., 2012), which makes it difficult to speculate about the biological significance of this phenomenon. Whether there is a relation with the reported capacity of fHbp to bind siderophores produced by other bacteria (Veggi et al., 2012) is questionable as fHbp synthesis is reduced in most strains under iron limitation when siderophore production is induced. A physiological role for the siderophore-binding capacity of fHbp has not been demonstrated.

The structure of fHbp has been determined by NMR and crystallography (Cantini et al., 2009; Schneider et al., 2009). The overall fold resembles those of HpuA and of the individual lobes of TbpB and LbpB and consists of a C-terminal β-barrel and an N-terminal taco-shaped barrel-like structure (Figure 5). Factor H consists of 20 complement control protein repeats, and of these, repeats 6 and 7 were found to bind fHbp with extensive interactions in co-crystals (Schneider et al., 2009). Interestingly, this binding site overlaps with the binding site for glycosaminoglycans to which factor H binds on mammalian cells to prevent complement activation.

#### Neisseria heparin-binding antigen (NHBA)

Like fHbp, NHBA (a.k.a. GNA2132) is a component of the Bexsero® vaccine. In spite of considerable sequence variability, particularly near the N terminus (Bambini et al., 2009), NHBA elicits broadly cross-reactive bactericidal antibodies that conferred passive protection in a rat model (Welsch et al., 2003; Giuliani et al., 2010; Serruto et al., 2010).

NHBA was shown to bind heparin and heparan sulfate (Serruto et al., 2010). It consists of two domains. The structure of the conserved C-terminal domain has been determined by NMR and shows a similar 8-stranded β-barrel as those in the lipoproteins described above (Figure 5) (Esposito et al., 2011). The structure of the N-terminal domain has not been solved; it was predicted to be an intrinsically unfolded polypeptide (Esposito et al., 2011). In between these domains, NHBA contains an arginine-rich segment that binds heparin and heparan sulfate by electrostatic interaction (Serruto et al., 2010). The binding of heparin and heparin-like molecules has several functions. First, heparin binding was shown to correlate with increased survival of an unencapsulated *N. meningitidis* strain in human serum. In an encapsulated strain, such effect of NHBA synthesis was not observed, presumably because of the dominant role of the capsule in mediating serum resistance. As heparin is known to interact with complement proteins, including factor H, C4b-binding protein and C1 inhibitor (Yu et al., 2005), it was suggested that heparin bound at the cell surface might impart serum resistance by its capacity to bind such complement-regulatory molecules (Serruto et al., 2010). Alternatively, it might form a negatively charged capsule-like structure resembling the classical polysaccharide capsule (Serruto et al., 2010). NHBA has also been shown to function as an adhesin by binding to heparan sulfate proteoglycans that are present on surface of epithelial cells (Vacca et al., 2016).

Besides heparin and heparin-like molecules, NHBA also binds other polyanionic structures, including DNA (Arenas et al., 2013a). *N. meningitidis* forms microcolonies on the epithelial surfaces in the nasopharynx (Sim et al., 2000). Extracellular DNA (eDNA) is an important component of the extracellular matrix of these biofilm-like structures, and it is essential in the initiation of biofilm formation in most clonal lineages (Lappann et al., 2010). NHBA binds eDNA and inactivation of the *nhbA* gene in these lineages impairs biofilm formation (Arenas et al., 2013a).

NHBA can be cleaved immediately upstream of the arginine-rich segment by the autotransporter protease NalP (see below). Consequently, a C-terminal fragment including the arginine-rich segment is released into the milieu (Serruto et al., 2010). This fragment is taken up by endothelial cells and accumulates in mitochondria, where it induces the production of reactive oxygen species. The resulting phosphorylation of the adherens junction protein VE-cadherin and its subsequent internalization leads to increased endothelial permeability (Casellato et al., 2014). Thus, the released fragment of NHBA may contribute to the extensive vascular leakage that is typical for meningococcal sepsis. The arginine-rich segment was essential for this role of the NHBA C-terminal fragment presumably by functioning as a mitochondrial targeting signal. A slightly smaller fragment that is released from NHBA by lactoferrin-mediated cleavage and that lacks the arginine-rich segment did not induce endothelial leakage (Casellato et al., 2014).

### Autotransporters

#### IgA protease

IgA protease is produced by both *N. meningitidis* and *N. gonorrhoeae*. The passenger domain consists of two subdomains, an N-terminal domain containing the protease activity and a polypeptide called the α-peptide; these domains are connected via a small γ-peptide (Pohlner et al., 1987). The passenger is connected to the β-domain via a linker peptide. The protease domain can be released into the extracellular milieu by autocatalytic cleavage at sites (PAPSP, PPSP, or PPAP) located in between the protease domain and the γ-peptide, between the γ-peptide and the α-peptide, and between the α-peptide and the linker peptide. The presence of the latter processing site is strain dependent (Roussel-Jazédé et al., 2014). Alternatively, the entire passenger including the linker peptide can be released after cleavage by the autotransporter protease NalP (see below) (van Ulsen et al., 2003). IgA protease cleaves human IgA1 at a site (TPPTPSPS), which resembles the autocatalytic processing sites and is located in the hinge region between the Fab and the Fc domains (Plaut et al., 1975). IgA protease does not cleave IgA2, which lacks such a cleavage site (Plaut et al., 1975). Cleavage of IgA1 may inhibit IgA-mediated agglutination and subsequent mechanical clearance of the bacteria in the nasopharynx. IgA protease has also been shown to cleave the lysosome-associated membrane protein LAMP1 (Hauck and Meyer, 1997), which was reported to promote bacterial survival within epithelial cells (Lin et al., 1997) and transcytosis across polarized epithelia (Hopper et al., 2000). In addition, IgA protease cleaves the vesicular membrane protein synaptobrevin II in chromaffin cells (Binscheck et al., 1995) and the human chorionic gonadotropin hormone (Senior et al., 2001), but the physiological implications are not clear. All these alternative substrates have a target site resembling the autocatalytic cleavage sites.

The α-peptide contains a variable number of nuclear localization signals (NLS), which are arginine-rich peptide segments (Pohlner et al., 1995; Roussel-Jazédé et al., 2014), and it was indeed demonstrated to target an attached reporter protein to the nucleus of eukaryotic cells (Pohlner et al., 1995). When NalP releases IgA protease with attached α-peptide from the bacterial cell surface, the IgA protease is targeted to the nucleus where it cleaves the p65/RelA component of transcription factor NF-κB, thus silencing the expression of several NF-κB-responsive genes, including those encoding interleukin 8 and the anti-apoptotic protein cFLIP (Besbes et al., 2015). This altered gene expression results in sustained activation of c-Jun N-terminal kinase and apoptosis. Interestingly, purified IgA protease without attached α-peptide has been shown to inhibit TNFα-induced apoptosis of monocytes, presumably by the observed cleavage of the TNF receptor II (Beck and Meyer, 2000).

When NalP is not expressed and the autocleavage site between the α-peptide and the linker peptide is absent, the α-peptide is exposed at the bacterial cell surface covalently connected to the β-domain. Since NLS are positively charged protein segments, they bind polyanions like eDNA, thus stimulating biofilm formation (Arenas et al., 2013a). The α-peptide also binds heparin (Roussel-Jazédé et al., 2014) and may have similar functions in conferring serum resistance and mediating adhesion as discussed above for NHBA. Whether the α-peptide also directly affects gene expression by binding DNA after it has been targeted to the nucleus of eukaryotic cells has not been investigated yet.

#### NalP

The *nalP* gene is found in the vast majority of *N. meningitidis* isolates, with the notable exception of invasive isolates of clonal complexes ST-269 and ST-461 (Oldfield et al., 2013). Its expression is prone to phase variation by slipped-strand mispairing at a poly-C tract in the coding region (Turner et al., 2002; van Ulsen et al., 2003). An analysis of its phase variation status revealed that approximately half of the strains were phased on, and no significant differences were found in this respect between carriage and invasive strains (Oldfield et al., 2013).

NalP is a subtilisin-like serine protease. Its passenger is released from the cell surface by autoproteolytic cleavage. However, after cleavage between the passenger and the β-domain, the passenger remains temporarily attached at the cell surface by an N-terminal lipid anchor (Roussel-Jazédé et al., 2013). In this position, it releases several autotransporters and lipoproteins from the bacterial cell surface. The release of these proteins has relevant consequences. As already discussed above, the NalP-mediated release of IgA protease with attached α-peptide and of the C-terminal part of NHBA from the cell surface stimulates apoptosis and increases the permeability of endothelial and epithelial cell layers. In contrast, when *nalP* is phased off, the α-peptide and complete NHBA are retained at the cell surface, which stimulates biofilm formation and adhesion and protects against host defenses. Thus, the absence of *nalP* expression appears to favor colonization and biofilm formation, whilst *nalP* expression may lead to dispersal of biofilms and invasion or spreading to a new host.

Another target of NalP is the LbpB component of the lactoferrin receptor (Roussel-Jazédé et al., 2010). LbpB is a very immunogenic protein, and its NalP-mediated release from the cell surface imparted protection against the complement-mediated killing by LbpB-specific bactericidal antibodies (Roussel-Jazédé et al., 2010). As discussed above, LbpB is not essential for the acquisition of iron from lactoferrin, although it may enhance the process by selecting holo-lactoferrin for binding to the receptor. The latter function may be lost when LbpB is cleaved from the cell surface by NalP, but this is compensated by protection against LbpB-specific bactericidal antibodies. Another function of LbpB discussed above, i.e., offering protection against the membrane-damaging effects of lactoferricin, is probably retained, even if the protein is released into the extracellular milieu. Thus, due to the phase-variable expression of *nalP*, a subpopulation of the bacterial cells will lose LbpB from the cell surface, and this subpopulation may be less efficient in iron acquisition but will have a competitive advantage when LbpB-specific antibodies are elicited in the host. It is noteworthy that in *N. gonorrhoeae*, which does not produce NalP because the gene is disrupted, the expression of *lbpB* itself is prone to phase variation (Anderson et al., 2003).

Besides bacterial proteins, NalP also cleaves at least one host protein, i.e., the complement factor C3. Thereby, it avoids complement activation and confers serum resistance (Del Tordello et al., 2014). Consistently, *nalP* expression was earlier reported to be upregulated during growth in an *ex vivo* human whole-blood model and to be essential for survival under these conditions (Echenique-Rivera et al., 2011b).

#### Two additional proteases: App and AusI

App and AusI (a.k.a. MspA) are both chemotrypsin-like serine proteases with homology to IgA protease (Abdel Hadi et al., 2001; van Ulsen et al., 2001, 2006; Turner et al., 2006). Whereas, an intact *app* gene is ubiquitous among *N. meningitidis* strains, the *ausI* gene is disrupted by premature stop codons in several strains, and its expression is prone to phase variation by the presence of a poly-C tract in the coding region (Martin et al., 2003; van Ulsen et al., 2006). Interestingly, an extensive study in mostly serogroup B and Y isolates revealed that the *ausI* gene was phased on in 90% of both invasive and carriage strains of serogroup B. As 33% phased on would be expected in the case of random on and off switching, this suggests an important role for AusI in the biology of serogroup B (Oldfield et al., 2013). Such a preference for the on phase of *ausI* was not found in the serogroup Y isolates.

App and AusI are both released from the bacterial cell surface by autoproteolytic cleavage. However, like for IgA protease, a larger form of these proteins with a C-terminal extension, called α-peptide, can be released by NalP-mediated cleavage (van Ulsen et al., 2003, 2006; Turner et al., 2006). The α-peptide of App contains two NLS similar to those in the α-peptide of IgA protease, but such sequences are not present in the α-peptide of AusI. Nevertheless, both proteins with attached α-peptides were shown to be targeted to the nucleus after being taken up by dendritic cells and to induce apoptosis, presumably by their capacity to bind histones and to cleave histone H3 (Khairalla et al., 2015). Uptake into the dendritic cells was shown to be mediated by the mannose receptor and the transferrin receptor 1 to which the proteases could be crosslinked.

Earlier, both App and AusI were reported to function as adhesins (Serruto et al., 2003; Turner et al., 2006), a function which is difficult to reconcile with their cleavage from the cell surface. Perhaps, the proteins remain temporarily associated with the cell surface before they are released. Heterologous expression of App and AusI in *E. coli* mediated adhesion of the bacteria to Chang epithelial cells, whereas variable adhesion to other cell lines was observed (Serruto et al., 2003; Turner et al., 2006). App-mediated adhesion to Chang cells could also be demonstrated in *N. meningitidis* (Serruto et al., 2003). The presence of a capsule did not seem to interfere with the adhesin function, suggesting that this large protein (~160 kDa) extends beyond the capsule to interact with its receptor on the Chang cells. However, deletion analysis suggested that the α-peptide, which is located close to the bacterial cell surface in uncleaved App and is unlikely to extend beyond the capsule, is the main determinant for adhesion (Serruto et al., 2003). Protease treatment indicated the involvement of a proteinaceous receptor on Chang cells, but it is not known whether the transferrin receptor 1 or the mannose receptor, which mediate uptake of App and AusI in dendritic cells (see above), are involved. App was also shown to mediate interbacterial aggregation on cell layers (Serruto et al., 2003); possibly, this is mediated by the binding of the NLS in the α-peptide to eDNA, analogous to the role of the α-peptide of IgA protease in biofilm formation (see above).

#### Surface-exposed classical autotransporters: AutA and AutB

AutA and AutB are two related, relatively small autotransporters of ~75 kDa including the β-domain. The *autA* gene is broadly distributed among *Neisseria* spp., whereas the presence of the *autB* gene is restricted to the pathogenic *Neisseria* spp., *N. meningitidis* and *N. gonorrhoeae* (Arenas et al., 2015a, 2016). Also some, but not all, strains of *Haemophilus influenzae* and closely related *Haemophilus* spp. contain an *autB* gene. Analysis of the *autB*-flanking sequences in both genera suggested that a common ancestor of the two pathogenic *Neisseria* spp. has acquired the gene from a *H. influenzae* strain by horizontal gene transfer (Davis et al., 2001; Arenas et al., 2016).

The expression of *autA* and *autB* is prone to phase variation by slipped-strand mispairing at AAGC nucleotide repeats located immediately downstream of the start codon (Peak et al., 1999). In addition, the *autA* gene was disrupted in 76% of the meningococcal genomes analyzed by premature stop codons, insertions and/or deletions (Arenas et al., 2015a) suggesting selection pressure against expression of the gene. However, the gene was phased on in ~33% of the strains with an intact *autA*, consistent with random on and off switching. In contrast to *autA, autB* was disrupted in only 8% of the *N. meningitidis* genomes analyzed (Arenas et al., 2016). Curiously, the gene is phased off in the vast majority of the genomes with an intact *autB*, and in only ~2% of the strains the gene could be expressed. This strongly suggests that, even if an intact *autB* is retained, its expression is under negative selection pressure probably already in the nasopharynx as no significant difference between invasive and carriage isolates was observed in this respect (Arenas et al., 2016).

The passenger domains of AutA and AutB are not proteolytically released, and they are exposed at the bacterial cell surface covalently attached to the β-domain (Arenas et al., 2015a, 2016). AutA expression was shown to cause bacterial autoaggregation (Arenas et al., 2015a), a trait associated with resistance to host immune defenses such as phagocytosis (Ochiai et al., 1993). Autoaggregation is mediated by mutual interaction of AutA proteins on neighboring cells. In addition, AutA was found to bind DNA, which acts as an adhesive between cells (Arenas et al., 2015a; Arenas and Tommassen, 2017). Autoaggregation also has consequences for the architecture of biofilms formed. Besides, AutA was reported to be a potent CD4^+^ T-cell and B-cell stimulating antigen (Ait-Tahar et al., 2000). Expression of *autB* was shown to enhance biofilm formation, and it retarded the passage of the bacteria through epithelial cell layers (Arenas et al., 2016). This suggests that AutB could have a role during host colonization.

#### Trimeric autotransporters: NadA and NhhA

NadA shows high sequence similarity to the well-known virulence factors YadA from *Yersinia enterocolitica* and UspA2 from *Moraxella catarrhalis* (van Ulsen et al., 2001). It is a component of the Bexsero® vaccine. However, the gene is present in only ~30% of *N. meningitidis* isolates with a lower prevalence in carriage strains (~15%) (Comanducci et al., 2002, 2004). Furthermore, based on sequence variability, two main groups of NadA variants can be discriminated (Bambini et al., 2014) with limited immunological cross-reactivity between the groups (Comanducci et al., 2004), and the expression levels are highly variable (Martin et al., 2003), which also may impact on vaccine efficacy (Comanducci et al., 2002). Expression levels are determined by phase variation at a TAAA tetranucleotide repeat region upstream of the *nadA* promoter (Martin et al., 2003), whilst also on/off switching can occur by phase variation at a poly-C stretch within the coding region of some *nadA* variants (Bambini et al., 2014). Expression of *nadA* is transcriptionally controlled by the repressor NadR (Schielke et al., 2009), which binds operator sequences upstream and downstream of the TAAA repeat region, the latter overlapping with the -10 region of the promoter (Metruccio et al., 2009). The metabolite 4-hydroxyphenylacetic acid was found to act as an inducer of *nadA* expression (Metruccio et al., 2009). This metabolite is secreted in human saliva, suggesting that *nadA* expression will be induced on the mucosal surfaces of the nasopharynx. Interestingly, upon derepression, the expression levels were similar independent of the number of TAAA repeats, indicating that the number of repeats determines the level of repression by NadR rather than the expression levels in the induced state.

NadA functions as an adhesin and invasin. Its heterologous expression in *E. coli* promoted adherence of the bacteria to and invasion of Chang epithelial cells, and this function was confirmed in *N. meningitidis* (Capecchi et al., 2005). NadA function could also be reconstituted in a *yadA* mutant of *Y. enterocolitica*, and it was shown that β1 integrins function as its receptor on host cells (Nägele et al., 2011). Curiously, NadA was also shown to bind the heat shock protein Hsp90, a normally cytoplasmic chaperone, and purified Hsp90 was shown to inhibit NadA-mediated adhesion and invasion *in vitro* (Montanari et al., 2012). The physiological significance of these observations is not clear.

**Figure 6:**
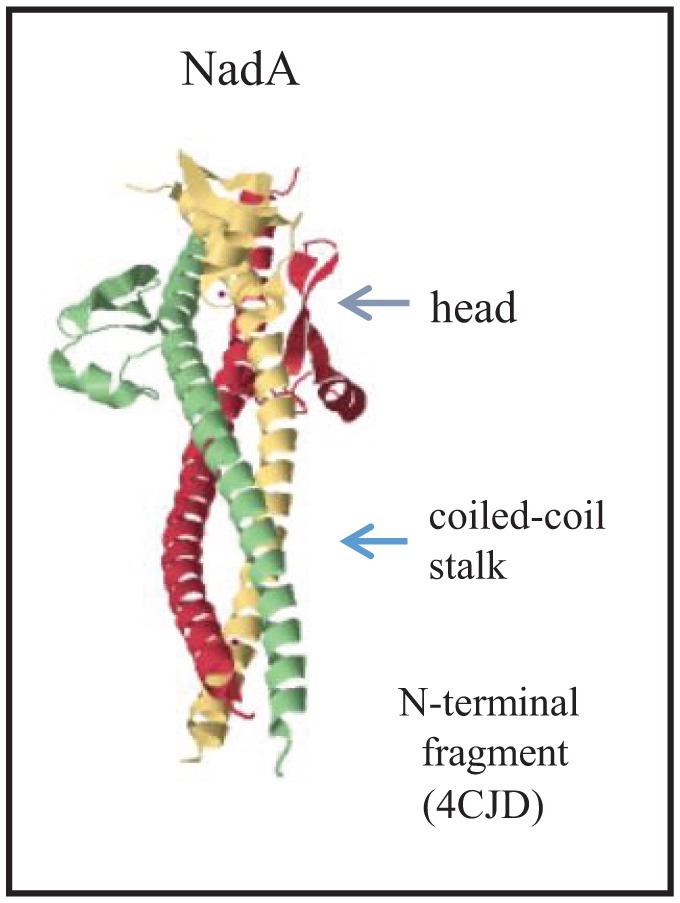
Structure of the passenger of the trimeric autotransporter NadA. The structure of the N-terminal part of the passenger is provided (Malito et al., 2014). Each subunit is colored differently, and the major structural domains of the complex are indicated. The PDB access code is provided in brackets.

Whilst the passenger domains of classical autotransporters usually form a long β-helical structure that displays globular functional domains, trimeric autotransporters have a completely different architecture consisting of a stalk domain that displays one or several head domains (Grijpstra et al., 2013). The stalk is a coiled-coil structure that extends from the α-helices that occupy the membrane-embedded β-barrel (Figure 1D). NadA has a similar overall architecture but is somewhat atypical in that a separate globular head domain is lacking (Malito et al., 2014). Instead, the coiled coils extend up to the N terminus and the head is formed by insertions of 36 residues that protrude from the stalk and form wing-like structures (Figure 6). The adhesin activity was mapped to the head region (Capecchi et al., 2005; Tavano et al., 2011), which is apparently sufficiently exposed from the bacterial cell surface to be functional also in encapsulated strains (Capecchi et al., 2005).

NhhA (a.k.a. Msf) was identified as a homolog of the adhesin Hia of non-typeable *H. influenzae* (Peak et al., 2000; van Ulsen et al., 2001), and the *nhhA* gene was found to be ubiquitously present in all *N. meningitidis* strains examined, although disrupted in some of them (Peak et al., 2000). Expression levels varied widely and were affected in one particular lineage, i.e., ST41/44, by an amino-acid substitution in the β-domain, which prevents trimerization and surface exposition of the passenger domain (Echenique-Rivera et al., 2011a). The function of NhhA as an adhesin was demonstrated after its heterologous production in *E. coli*, which mediated adherence of the bacteria to epithelial cell lines, and was confirmed after constructing an *nhhA* mutant in an encapsulated *N. meningitidis* strain (Scarselli et al., 2006). It was demonstrated that purified NhhA and NhhA-producing cells bind the extracellular matrix components laminin and heparan sulfate (Scarselli et al., 2006). In addition, NhhA was shown to bind activated vitronectin, a complement regulatory protein, and, thus, to impart serum resistance to the bacteria by inhibiting the formation of the membrane-attack complex (Griffiths et al., 2011). Consistently, an *nhhA* mutant was less virulent and showed reduced survival in blood after intraperitoneal challenge of mice (Sjölinder et al., 2008). Expression of the protein appeared to be essential also for the colonization of the nasopharyngeal mucosa and for disease development after intranasal challenge in a murine model of meningococcal disease. The reduced colonization levels are probably related to the *in vitro* observations that wild-type bacteria are more resistant than the *nhhA* mutant to phagocytosis (Sjölinder et al., 2008) and that NhhA induces apoptosis of macrophages (Sjölinder et al., 2012). In addition, immune modulatory activities of NhhA were reported (Wang et al., 2016). NhhA blocked differentiation of monocytes into dendritic cells and induced their differentiation into macrophages that failed to produce proinflammatory mediators but produced anti-inflammatory responses. These differentiated macrophages were highly efficient in eliminating the bacteria. Hence, this response prevents dissemination and sustains asymptomatic colonization (Wang et al., 2016).

### The two-partner secretion (TPS) system

The substrates of the TPS systems, TpsA, are very large proteins (>2,000 amino-acid residues), which, like most classical autotransporters, display a β-helical conformation (Jacob-Dubuisson et al., 2013). *N. meningitidis* strains can produce up to five different TpsA proteins (van Ulsen and Tommassen, 2006). The genes for one TPS system are widely distributed among *N. meningitidis* isolates, whereas the genes for other TPS systems are associated with invasive clonal complexes (van Ulsen et al., 2008). Only the function of the conserved TpsA, a.k.a. HrpA, has been investigated, particularly in strains that produce this protein as the only TpsA. Substrates of TPS systems can have various, often virulence-related functions. Several functions have been attributed to the meningococcal TpsA, including a role in intracellular survival and escape from infected cells (Talà et al., 2008), adhesion to epithelial cell lines (Schmitt et al., 2007), and biofilm formation (Neil and Apicella, 2009). More recently, it was demonstrated that TpsA is involved in interbacterial competition in a process called contact-dependent growth inhibition (CDI) (Arenas et al., 2013b). CDI was originally described in *E. coli* and has evolved to inhibit the growth of closely related bacteria, usually of the same species, in niche competition (Aoki et al., 2005). In the proposed model, the surface-exposed TpsA interacts with a receptor in the outer membrane of a target cell, after which a small C-terminal part of TpsA of ~300 amino-acid residues is proteolytically released and transported into the target cell, where it exerts one of different toxic activities, e.g., by functioning as a DNase or an RNase (Aoki et al., 2010). The producing cells themselves are protected against this toxic activity by the production of a small immunity protein, here called TpsI. TpsA proteins with CDI activity are polymorphic toxins, i.e., comparison of their sequences in different strains revealed that they can display a wide variety of toxic domains at their C terminus, each of which is associated with its own cognate TpsI (Aoki et al., 2010).

**Figure 7:**
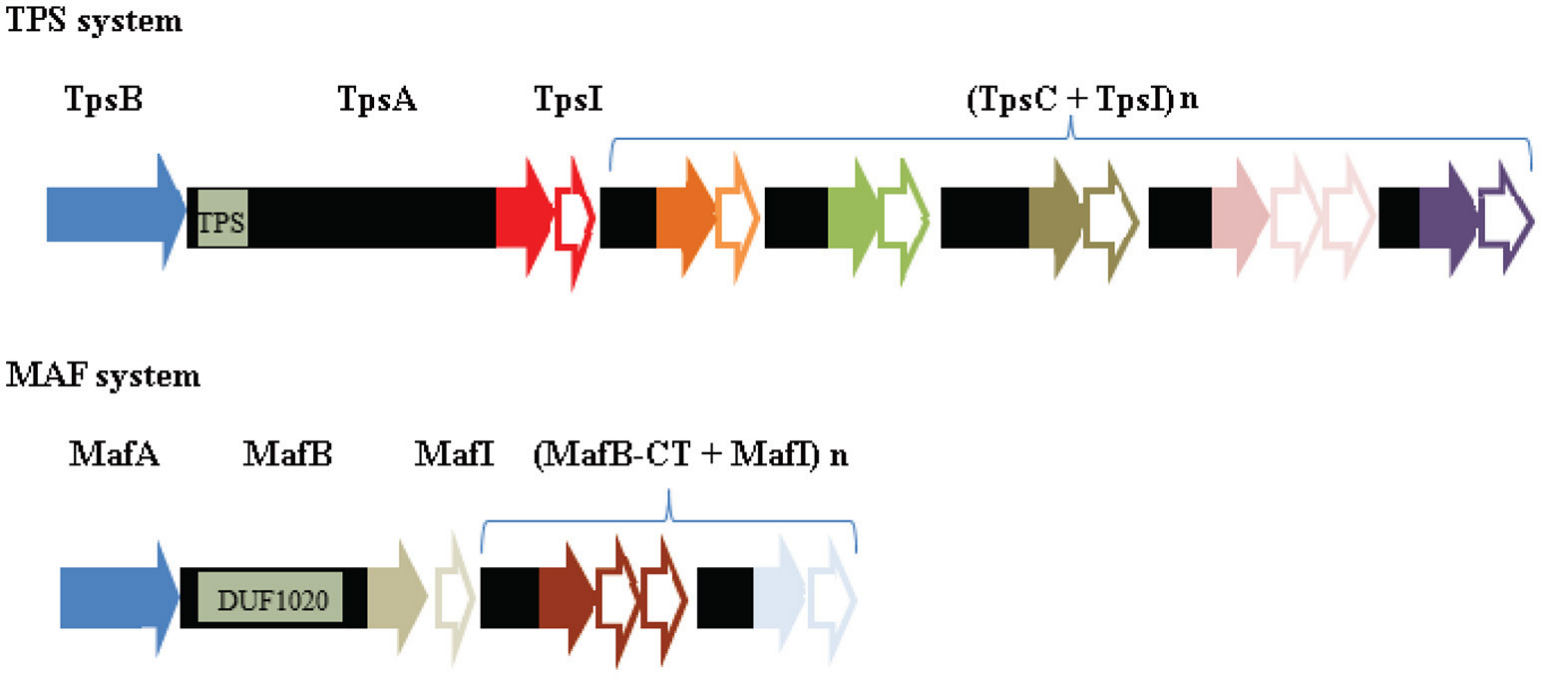
Comparison of the genetic organization of the genes encoding a TPS system and a MAF system. In the top panel, the genetic island encoding the TPS system of strain B16B6 is depicted (Arenas et al., 2013b). TPS islands consist of genes encoding the transporter TpsB (blue), the secreted toxin TpsA, an immunity protein TpsI, and a variable repertoire of *tpsC* cassettes interspersed with genes encoding immunity proteins. The position of the gene segment of *tpsA* that encodes the TPS domain, which is recognized by TpsB, is indicated. The segments of *tpsA* and *tpsC* cassettes that encode toxic domains are colored, with different colors indicating different toxic domains. The *tpsI* genes encoding the cognate immunity proteins are indicated as open arrows with the same colors. In the bottom panel, the genetic island MGI-1 of B16B6 is depicted (Arenas et al., 2015b). The MGI islands consist of genes encoding MafA, the secreted toxin MafB, an immunity protein MafI, and a variable repertoire of *mafB-CT* cassettes interspersed with genes encoding immunity proteins. The position of the gene segment that encodes the conserved DUF1020 domain in MafB is indicated. Sequences encoding toxic domains and cognate immunity proteins are indicated as in the TPS island. Note that no evidence for MafA representing the transporter of MafB has been reported so far.

The genes for the conserved TPS system of *N. meningitidis* are organized in an operon (Figure 7). Downstream of the *tpsI* gene, a variable number of 5′ truncated *tpsA*-related genes are located, called *tpsC* cassettes. In their 5′ end, the *tpsC* cassettes show homology with a segment immediately upstream of the 3′ end encoding the toxic domain of *tpsA*. At their 3′ end, however, the *tpsC* cassettes encode different toxic domains (Figure 7). Thus, the *tpsC* cassettes provide a broad repertoire of toxic domains and each of them is associated with a cognate *tpsI* gene to impart immunity to the producer. However, the products of these *tpsC* cassettes, if expressed, cannot be secreted because they lack an N-terminal signal sequence and a TPS domain, required for recognition by the Sec system and the TpsB protein, respectively. It was proposed that the 3′ end of *tpsA* along with the downstream *tpsI* can be replaced by *tpsC* cassettes and the cognate *tpsI* through genetic recombination, thus resulting in the production of a TpsA with a different toxic domain (Arenas et al., 2013b). Examination of the 3′ end of the *tpsA* genes in available genome sequences indeed suggested a high rate of recombination between *tpsA* and *tpsC* cassettes (van Ulsen and Tommassen, 2006). However, a detailed analysis of the 3′ end of the *tpsA* gene in large panels of meningococcal isolates from the same lineage collected over several decades from different parts of the world revealed a very low rate of exchange (Arenas et al., 2013b). It was suggested that the availability of a large collection of *tpsI* genes might be more important for the bacteria than the ability to replace the toxic domain of TpsA by a variety of others encoded by the *tpsC* cassettes.

### The T1SS

FrpC and related proteins (some called FrpA) are the substrates of the meningococcal T1SS; they are produced under iron limitation (Thompson and Sparling, 1993; Thompson et al., 1993a,b). FrpC-like proteins are large proteins of 120–200 kDa. They are members of the RTX family of proteins and differ largely with respect to the number of glycine-rich calcium-binding repeats (see above); in addition they show considerable sequence variability in a small N-terminal domain of ~350 amino-acid residues (van Ulsen and Tommassen, 2006), possibly due to immune pressure. The RTX family includes important cytotoxins in several pathogens (Linhartová et al., 2010). However, *N. meningitidis* mutants lacking functional *frpC* genes or the T1SS machinery were not attenuated in animal infection models (Klee et al., 2000; Forman et al., 2003).

After secretion, the FrpC protein is autocatalytically cleaved at an Asp-Pro bond at physiological concentrations of calcium ions (Osička et al., 2004). The N-terminal part of ~45 kDa is retained at the bacterial cell surface by binding FrpD with high affinity (Prochazkova et al., 2005). FrpD is a cell-surface-exposed lipoprotein that is encoded by the same operon as FrpC. Its structure is different from those of the surface-exposed lipoproteins discussed above, although it also has a high β-sheet content, which may be relevant for its transport to the cell surface (Figure 5) (Sviridova et al., 2017). During autocatalytic processing, the released carboxyl group of the C-terminal aspartate of the N-terminal FrpC fragment forms a covalent linkage with an adjacent ε-amino group of a lysine generating a new Asp-Lys isopeptide bond (Prochazkova et al., 2005). In this way, the N-terminal FrpC fragment may be covalently linked to membrane proteins on the host epithelial cells (Sviridova et al., 2017). Thus, together with FrpD, the N-terminal fragment of FrpC may function as an adhesin, mediating the adhesion of the bacteria to epithelial cells.

### Other secretion systems and secreted proteins

Besides the secretion systems discussed above, some *N. meningitidis* strains contain a type 4 secretion system (T4SS). The genes for a T4SS were first identified in gonococcal strains on a genomic island called gonococcal genomic island (Dillard and and Seifert, 2001), and the system was shown to mediate the secretion of single-stranded DNA (Hamilton et al., 2005). Subsequently, it was found that also some meningococcal strains contain genes for the T4SS (Snyder et al., 2005; Woodhams et al., 2012). However, in most of these strains, these genes are disrupted, and those strains with a complete T4SS do not appear to exhibit increased DNA release. As no function has been assigned to the T4SS in *N. meningitidis*, it won't be discussed here in detail. *N. meningitidis* also produce type IV pili, which are retractile pili involved in adhesion to host cells, interbacterial interaction, DNA uptake, and surface motility. These surface organelles are topic of several focused reviews to which we refer (e.g., see Berry and Pelicic, 2015; Mayer and Wong, 2015). In this section, we focus on a number of secreted proteins for which the secretion mechanism has not yet been solved.

#### MafB

Like the TpsA proteins, the MafB proteins are polymorphic toxins that display a small variable toxic domain at their C terminus (Arenas et al., 2015b; Jamet et al., 2015). The *mafB* genes are localized on the chromosome on genomic islands designated Maf Genomic Islands (MGI) (Jamet et al., 2015). Up to three MGI can be present in a single meningococcal strain. The gene organization on the MGI is reminiscent of the loci encoding the TPS systems (Figure 7). The *mafB* genes are usually flanked by a *mafA* and a *mafI* gene. The *mafI* gene encodes a small immunity protein, which has been shown to interact with the toxic domain of MafB and to neutralize its toxic activity (Arenas et al., 2015b; Jamet et al., 2015). Indeed, MafB producers were shown to inhibit the growth of congeners that do not produce the cognate immunity protein. Downstream of the *mafI* gene, a variable number of 5′ truncated *mafB*-related genes are located, called *mafB-CT* cassettes (Figure 7). Like the *tpsC* cassettes, these *mafB-CT* cassettes offer a reservoir of alternative toxic domains, which could potentially be displayed at the C terminus of MafB after a genetic recombination event.

Apart from the presence of a toxic domain, MafB proteins do not show any similarity with TpsA proteins. They are synthesized with a signal sequence for transport across the inner membrane via the Sec machinery. Immediately after the signal sequence, all MafB proteins contain a conserved domain of unknown function, DUF1020, followed by the C-terminal toxic domain (Figure 7). In some MafB proteins, a Hint domain, which is expected to mediate protein splicing (Amitai et al., 2003), separates the DUF1020 from the toxic domain. How MafB is translocated across the outer membrane is unclear. Considering the parallelism with the TPS loci (Figure 7), MafA would be an obvious candidate for a protein involved in the secretion process. Earlier studies in *N. gonorrhoeae* indicated that MafA is an adhesin that binds host glycolipids (Paruchuri et al., 1990). Accordingly, these proteins were designated members of a multiple adhesin family (maf). MafA doesn't show any similarity with TpsB. It is produced with a signal sequence containing a lipobox motif, suggesting it's a lipoprotein, and secondary structure predictions didn't suggest a β-barrel structure, which is difficult to reconcile with its possible role as a transporter (Arenas et al., 2015b). Indeed, MafB was found in the extracellular medium when expressed in a *Neisseria cinerea* mutant lacking MafA (Jamet et al., 2015), suggesting that MafB secretion is independent of MafA. Furthermore, MafB was not secreted when produced in *E. coli* suggesting that it is not a novel type of autotransporter and that the transport mechanism involved is not present in *E. coli* (Jamet et al., 2015). It was suggested that MafB may be released with OMVs, which are abundantly shed from the surface in *N. meningitidis* (Jamet et al., 2015).

#### T and B cell-stimulating protein B (TspB)

The name TspB, for T- and B-cell-stimulating protein B, may be a misnomer as evidence for such a function has, to the best of our knowledge, never been published. TspB was identified as a protein that binds immunoglobulins (Ig) (Müller et al., 2013). The protein is encoded by a prophage that is associated with invasive-disease isolates (Bille et al., 2005; Müller et al., 2013), and its synthesis is induced in the presence of human serum (Müller et al., 2013). Up to four copies of the *tspB* gene can be found in a genome.

The TspB proteins contain variable sequences at the N- and C-terminal ends and a conserved core region followed by a variable proline-rich segment (Müller et al., 2013). The core region of TspB binds the Fcγ region of IgG2. Ig-binding proteins are produced by several human pathogens, and they prevent the activation of complement. Indeed, TspB was shown to contribute to the survival of *N. meningitidis* in normal human serum by inhibiting IgM-mediated activation of the classical complement pathway (Müller et al., 2015). Since TspB binds IgM only poorly (Müller et al., 2013), this effect is probably indirect. TspB was found to bind DNA and to the form a biofilm-like matrix consisting of TspB, IgG, and DNA surrounding bacterial aggregates (Müller et al., 2013, 2015). Possibly, the presence of IgG in the matrix stimulates the non-productive activation of complement far away from the bacterial cell surface.

The secretion mechanism of TspB remains unclear. It could be released as a minor coat protein of filamentous phage particles, which was demonstrated to be mediated by the secretin PilQ, a protein also responsible for extrusion of type IV pili from the bacteria (Bille et al., 2005). However, in the studies of Müller et al. (2013), TspB appeared not to be associated with phage particles. The protein is produced with an N-terminal signal sequence for Sec-mediated transport across the inner membrane and a hydrophobic region near the C terminus that should act as a stop-transfer signal and anchor the protein in the membrane. Thus, possibly, the protein is only released after cell lysis.

## Concluding remarks

The quest for novel vaccine antigens has enormously stimulated research into cell-surface-exposed and secreted proteins in *N. meningitidis*. These investigations have not only resulted in the development of two new registered vaccines, but they have also uncovered the functions of many of these proteins, which have roles in host-pathogen interactions, including adhesion, invasion, and immune evasion, in nutrient acquisition, and in interbacterial interactions, including biofilm formation and competition. Thus, these studies have provided important novel insights into biology of the meningococcus. In addition, they have led to the discovery of new transport mechanisms and machineries of general (micro)biological significance, such as the BAM and SLAM discussed above and the LPT machinery for the transport of lipopolysaccharides (Bos et al., 2004). Because they are essential in most Gram-negative bacteria and cell-surface exposed, particularly the integral OMPs of the BAM and LPT systems are attractive targets for the development of new antimicrobials (Srinivas et al., 2010; Urfer et al., 2016).

With respect to the transport mechanisms, there is still much to be learned. For lipoproteins, for example, it will be important to establish the sorting rules in *N. meningitidis*, which will help to predict on the basis of the sequence whether a lipoprotein is transported to the cell surface or is retained at the periplasmic side of either the inner or the outer membrane. In the MAF system, it is still unknown how MafB is transported to the cell surface, and how its toxic domain is cleaved off and imported into the target cell. Molecular insights in these mechanisms could lead to novel antimicrobial therapies.

Competition between different meningococci is a new field of study. A relatively large part of the rather small genome is occupied by genes encoding toxins and their cognate immunity proteins and transporters. It should be noted that CDI activity has, so far, only been demonstrated in competition assays between wild-type strains and their mutant derivatives lacking specific immunity proteins. What happens in a competition between two wild-type strains that express different toxins and immunity proteins is unknown. Although colonization by different strains has been demonstrated in carriage studies, it was very rare and detected in only ~1% of the carriers (Caugant et al., 2007), suggesting that competition in the nasopharynx may be very effective. In contrast, such competition was recently not observed *in vitro* in dual-strain biofilm-formation experiments (Pérez-Ortega et al., 2017).

It is evident that many components of the secretome exert similar functions. It is particularly remarkable how many of them function as adhesins. Some redundancy in function is to be expected considering the high capacity of the meningococcus to change its cell surface, e.g., by phase variation, presumably as a mechanism to escape from the immune response of the host. Thus, one protein may take over the function of another one that is switched off. However, adhesins may also work in combination or sequentially or target other receptors and, thereby, other host cells. It is also evident that many secreted proteins have multiple functions, which is conceivable considering their large size. In addition, the secreted proteases may have multiple targets and, thus, interfere with the host's metabolism and immune response in multiple ways. It is likely that new functions of the secretome will be uncovered in the coming years. In this respect, it is important to note that most functions were discovered so far in *in vitro* systems. The development of transgenic mouse models for nasopharyngeal colonization of *N. meningitidis* (Joshwich et al., 2013) certainly opens new avenues for understanding the role of secretome components in host-pathogen interactions, although limitations will persist considering the large number of host-specific components involved.

## Author contributions

All authors listed, have made substantial, direct and intellectual contribution to the work, and approved it for publication.

### Conflict of interest statement

The laboratory of JT has received research funding from GlaxoSmithKline Biologicals. The other author declares that the research was conducted in the absence of any commercial or financial relationships that could be construed as a potential conflict of interest.
